# Mindfulness meditation, time judgment and time experience: Importance of the time scale considered (seconds or minutes)

**DOI:** 10.1371/journal.pone.0223567

**Published:** 2019-10-18

**Authors:** Sylvie Droit-Volet, Magali Chaulet, Frederic Dutheil, Michaël Dambrun

**Affiliations:** 1 Université Clermont Auvergne, CNRS, Lapsco (UMR 6024), Clermont-Ferrand, France; 2 Université Clermont Auvergne, CNRS, Lapsco (UMR 6024), Physiological and Psychosocial Stress, University Hospital of Clermont-Ferrand, CHU Clermont-Ferrand, Preventive and Occupational Medicine, WittyFit, Clermont-Ferrand, France; Radboudumc, NETHERLANDS

## Abstract

This manuscript presents two studies on the effect of mindfulness meditation on duration judgment and its relationship to the subjective experience of time when the interval durations are on the second or the minute time scale. After the first 15 minutes of a 30-min meditation or control exercise, meditation-trained participants judged interval durations of 15 to 50 s or 2 to 6 min, during which they performed either a mindfulness meditation exercise or a control exercise. The participants’ scores on the self-reported scales indicated the effectiveness of the meditation exercise, as it increased the level of present-moment awareness and happiness and decreased that of anxiety. The results showed an underestimation of time for the short interval durations and an overestimation of time for the long intervals, although the participants always reported that time passed faster with meditation than with the control exercise. Further statistical analyses revealed that the focus on the present-moment significantly mediated the exercise effect on the time estimates for long durations. The inversion in time estimates between the two time scales is explained in terms of the different mechanisms underlying the judgment of short and long durations, i.e., the cognitive mechanisms of attention and memory, respectively.

## 1 Introduction

In recent years, there has been an explosion of interest in meditation. This enthusiasm for meditation results from the abundance of studies demonstrating its benefits for human beings. Meditation practice does indeed increase the feeling of well-being [1,2], reduces negative affects (anxiety, depression) [3,4,5,6], and enhances some attentional skills [7,8,9,10]. The practice of meditation also changes the relationship to time. However, the relationship between time and meditation remains a mystery, because it is largely under-investigated [11].

The practitioners of different meditative techniques report the feeling of being outside time when they meditate, as if time no longer exists [12,13]. This “timelessness” is often accompanied by the subjective experience of an acceleration of the passage of time during meditation, when the passage of time judgment (PoT judgment) is assessed with a self-reported scale indicating how quickly time seems to pass [14]. This feeling that time passes faster during meditation has been recorded in long-term meditators [15,16], and even in students practicing a mindfulness meditation exercise for the first time [17]. People who are more mindful also feel that time passes more quickly in a timing task [18]. The question that may be raised is: What does this self-reported feeling of the passage of time mean exactly? Is this PoT feeling linked to the judgment of durations? To try to answer these questions, recent studies have begun to experimentally examine the effects of meditation on the judgment of durations.

Several studies have reported no difference in the judgment of short durations between long-term meditation practitioners and control subjects in a wide series of temporal tasks [19,20,21,22]. In addition, the few studies which have detected a difference have indicated better temporal judgments in meditation practitioners without any greater time distortions. Schötz et al. [23] found that experienced meditators were more accurate and precise in their time judgments. Wittmann et al. [24] observed higher temporal accuracy and precision in people who scored higher for trait-mindfulness on a personality scale. In the same way, Droit-Volet, Fanget and Dambrun [25] observed an increase in sensitivity to time after a mindfulness meditation exercise (see also [26]). Therefore, time distortions in duration judgment tasks, which might be expected on the basis of self-reported experience of the acceleration of the passage of time, are not observed in mindful people, in experienced meditators, or just after a mindfulness exercise.

Some studies have therefore directly tested the judgment of durations during a mindfulness meditation exercise in which the state of consciousness is altered. Droit-Volet and Heros [22] administered short auditory stimuli (0.8, 1.2, 1.6, 2 s) during the last 15 minutes of a long mindfulness meditation session of 30 minutes. The results did not show any difference between the temporal judgments of experienced meditators and students with no previous exposure to meditation. All the participants underestimated the stimulus durations presented during the meditation exercise compared to those presented during a control exercise. Droit-Volet et al. [16] obtained the same underestimation of time during a meditation task compared to a control task when the participants did not have to judge stimulus durations but, instead, the temporal intervals (15, 30, 60 s) that had elapsed. This shortening of interval durations was observed with different mindfulness techniques (i.e. body scan, breathing meditation), and compared to different attentionally demanding control tasks. Despite a certain inter-individual variability, Glicksohn et al. [15] also observed a subjective shortening of stimulus durations (4, 8, 16, 32 s) when experienced meditators were placed in an altered sensory environment (i.e. whole body perceptual deprivation chamber), as was indicated by the longer durations that they produced in the temporal production task used.

The results of studies indicating an underestimation of durations during a meditation exercise have logically been explained in terms of attentional processes [16,22]. According to attentional models of the internal clock [27,28], the subjective duration depends on the amount of attentional resources allocated to the timekeeper (clock). The smaller the amount of attentional resources allocated to time, the smaller the number of time units counted by the timekeeper is, and the shorter time is estimated to be. The predictions of attentional-clock models have been validated in a wide series of studies using a dual-task or attentional interference paradigm [29,30]. Therefore, the shortening of estimated duration observed during a meditation exercise should result from the fact that this specific exercise captures attention more than most attentional tasks do.

However, the studies on the judgment of durations during a meditation exercise have examined only short durations (< 60 s), i.e. a time scale which does not correspond to the time experience reported by meditators. Although the temporal span considered by meditators when they report an acceleration of the passage of time is not clearly defined, it likely covers a period of time longer than 60 s, i.e., the entire period of the exercise, or at least several minutes. There are only two studies which have examined the effect of a meditation exercise on the judgment of such long temporal intervals, i.e., 13 minutes in Thönes and Wittmann’s [17] study and 5 minutes in Sucala and David’s study [31]. The first study observed that duration judgment was more accurate with a mindfulness meditation exercise (body-scan) than with a control exercise (relaxing to music), while the second study found no meditation effect on duration judgments. In addition, the passage of time was judged to be faster in the first study and slower in the second one. The inconsistency in the results of these two studies may be due to the use of different methods. In addition, they used a retrospective time judgment task which was different from the prospective time judgment task used in the studies reported above. Unlike in the prospective time judgment task, the participants in the retrospective time judgment task are not informed that they will have to judge time. The aim of this present study was thus to test the effect of a meditation exercise on the prospective time judgment of long interval durations of several minutes compared to that of shorter interval durations.

Only a few studies have examined the judgment of long durations of several minutes. It has nevertheless been suggested that the mechanisms involved in the judgment of long durations are different from those involved in the judgment of short durations [32,33]. The judgment of long durations would be largely based on memory processes, similar to those observed in the retrospective judgment of durations, and the judgment of short durations on the functioning of a timekeeper (internal clock system) that demands attentional resources. According to memory-based models of the *retrospective* judgment of durations [34,35,36], time estimates are a function of the amount of non-temporal information stored and retrieved in memory, namely the characteristics of the experiencer (emotion), the events (number, complexity) or the activity (effortless, attentionally demanding) performed during the time period [36,37]. The more attentionally demanding the activity performed during the temporal interval is, the longer the elapsed duration is retrospectively judged to be [36,37]. Consequently, whether the meditation exercise is an attentionally demanding task, we can assume that the practice of a meditation exercise should result in a temporal underestimation for short interval durations and a temporal overestimation for long interval durations of several minutes.

In addition, some recent studies have indicated that awareness of the passage of time (PoT judgment) and duration judgment are dissociated on short time scales, but linked to each other on long time scales of several minutes. Droit-Volet and her collaborators found that the awareness of the speed of the passage of time was a significant predictor of duration judgments for long intervals of several minutes [33,38,39]. In addition, the best predictors of the PoT judgment were the emotion and the activity—difficult to achieve or requiring attention—experienced by the participants during the long temporal interval to be estimated. The different links between the awareness of the speed of the passage of time and the duration judgment for different temporal scales reinforce the idea that different mechanisms underlie the judgment of short and long durations. In the present study, we therefore examined the effect of a meditation exercise on both the judgment of durations and the judgment of the passage of time for both short and long interval durations.

In our study, the participants, who had been trained in the practice of meditation, therefore had to judge interval durations belonging in two duration ranges, that of seconds and that of minutes, during either a mindfulness meditation exercise or a control exercise. The judgment of the passage of time during these exercises was also assessed. As in the procedure used by Droit-Volet et al. [16], the participants performed a temporal task during the last 15 minutes of a long 30-minute exercise. In addition, several studies have shown that the most important criteria determining the effectiveness of a meditation exercise are its effects on present awareness, anxiety and happiness. Indeed, the practice of meditation increases the awareness of the present moment and the feeling of happiness and decreases the anxiety level [2,6,40,41]. In our study, we thus also assessed these three psychological dimensions on self-reported scales. The participants filled in the scales before and after the meditation/control exercise as well as before the training phase, since meditation training can modify these dimensions. Our hypothesis was therefore that performing a meditation exercise should produce distortions in interval duration judgments compared to performing a control exercise. However, this distortion should take the form of a temporal underestimation for short durations in the seconds range, and a temporal overestimation for longer durations in the minutes range. In addition, we can assume that there will be a significant relationship between the feeling that time passes faster during meditation and the judgment of durations for the temporal intervals of several minutes, but not for those of a few seconds.

## 2 Experiment 1

### 2.1 Method

#### 2.1.2 Participants

The sample consisted of 60 participants (14 men and 46 women; Mean age = 20.32, SD = 2,64). They were undergraduate psychology students at the University of Clermont Auvergne and received course credits in return for their participation. They signed a written consent form to participate in this study, which was approved by the Sud-Est VI statutory Ethics committee (CPP) (2016/CE 102), according to the French law. In this consent form, they stated that they would perform the exercises correctly and seriously, knowing that they were able to withdraw from the study at any time.

#### 2.1.3 Material

During the exercise, each participant lay on his or her back on a floor mat, eyes closed, in an experimental room of the university laboratory. The experimenter sat at a table behind the participant with two computers. One of these was used for the audio recording used in the 30-min exercise, and the other one for the temporal task, which was controlled by the E-prime software. A sound (LA, 440 Hz) lasting for 300 ms indicated the beginning and the end of the interval duration to be judged. The participants gave their temporal responses orally to avoid motor action, and the experimenter recorded their responses.

There were two audio exercises, one for the mindfulness meditation exercise and the other for the control exercise. In the meditation exercise, the participants followed, as closely as possible, a guided body-scan exercise in which they focused attention on different parts of the body. In the control exercise, the activity consisted of listening to a series of poems randomly taken from a list of 10 poems called “Paroles” written by the French poet Jacques Préverts [42] (for the same material, see [16]).

#### 2.1.4 Self-reported scales

**Fig 1:**
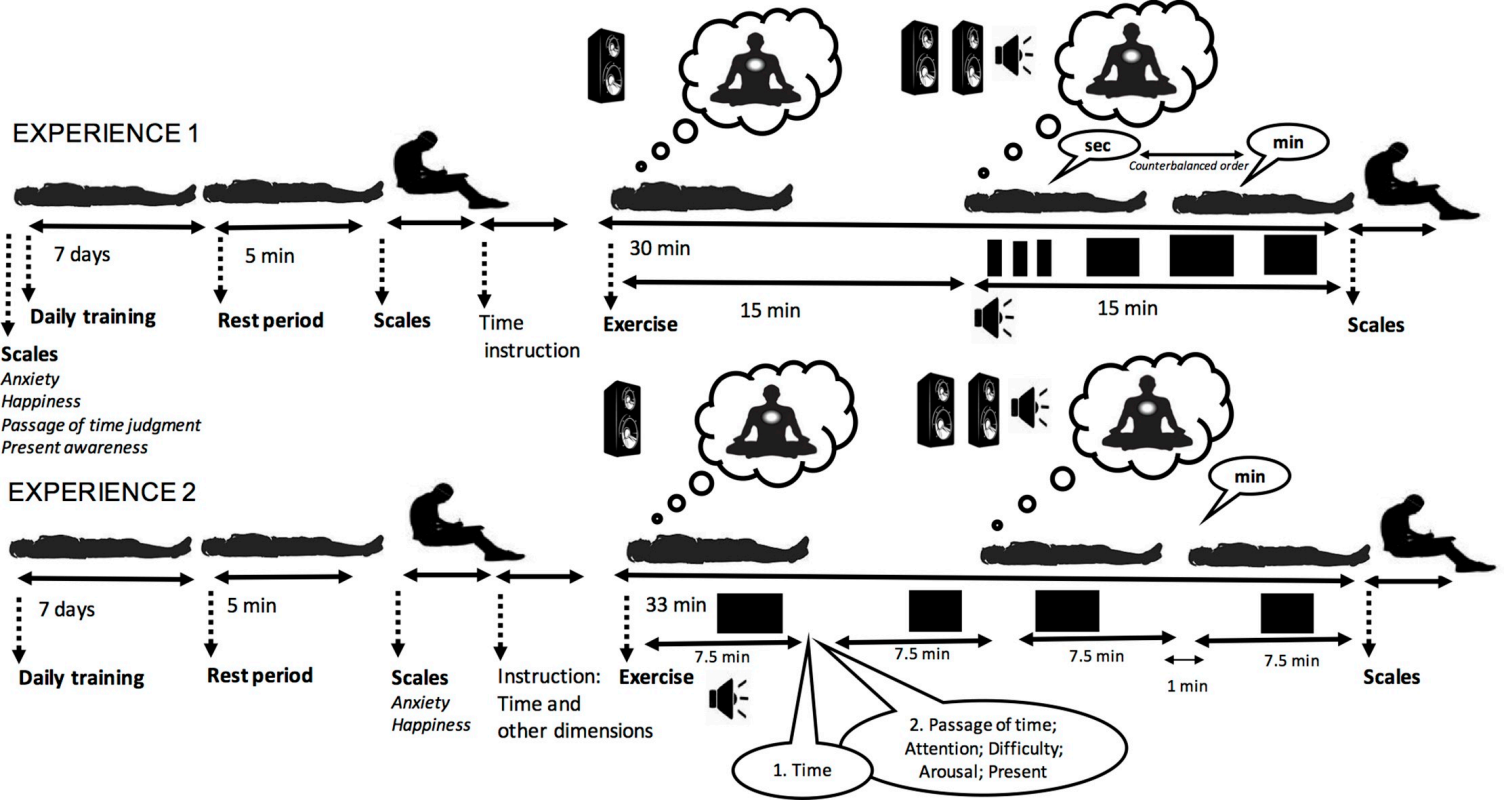
Experimental design. Schematic illustration of the procedure used in Experiments 1 and 2.

Four different self-reported scales were used with a random presentation order. These scales were filled in at 3 different times: (1) before the 7-day training (pre-training), (2) before the 30-min exercise (pre-exercise) and (3) after the exercise (post-exercise), i.e., after the session had been completed (Fig 1). At pre-training and pre-exercise, the participants completed the scales after 5 min of rest, during which they lay on the floor mat with their eyes closed.

The first scale was used to measure the level of anxiety. This was the six-item short-form of the Spielberg State Anxiety Inventory (STAI) [43]. The reliability of this scale was satisfactory as indicated by Cronbach’s alpha (pre-training: α = .831; pre-exercise: α = .829, post-exercise: α = .828). The second scale was the Subjective Authentic Durable Happiness Scale (SA-DHS), which measures the level of happiness [44]. This scale consists of 13 items (e.g., happiness, bliss, serenity) assessing authentic and lasting happiness. For each item of these two scales, the participants gave their responses on a continuous 14-cm line, going from “do not agree” to “totally agree”. The reliability of this scale was also good (pre-training: α = .951; pre-exercise: α = .968, post-exercise: α = .978).

Two other scales (questions) were used to assess the subjective experience of time. Both of these used the same continuous 14-cm line. The first scale went from “time is passing slowly” to “time is passing fast” and assessed the subjective judgment of the speed of the passage of time (PoT judgment). The second scale went from “low awareness of the present” to “high awareness of the present” and assessed the participant’s feeling of being focused more on the present moment than on the past/future (Present awareness).

#### 2.1.5 Procedure

The participants were randomly assigned to one of two experimental groups: meditation and control (30 participants per group). The procedure (Fig 1) was similar for the two groups, except for the type of exercise performed (mindfulness body-scan exercise or listening to poems). Before the test session in the laboratory, the participants received 11 min of guided at-home training for 7 days. Each day, they therefore performed an 11-min exercise at home, lying down on their backs on a floor mat with their eyes closed. The participants in the meditation group followed the instruction to focus their attention on the different parts of the body, while those in the control group listened to poems. The experimenter explained and demonstrated the different exercises to the participants beforehand. After each daily training session, the participants also completed a follow-up notebook.

The test session began with a 5-minute rest period (lying down, eyes closed), which was immediately followed by the completion of the scales. The participants then performed their group-specific exercise (meditation vs. control) for the same 30-min duration, still lying down with their eyes closed. After 15 minutes of exercise, the inter-beep intervals were presented. The participants were given two series of temporal intervals: one in the range of seconds and the other in that of minutes. The order of the two temporal series was counterbalanced so that one group of participants started with the short and the other with the long durations. There were 3 trials per temporal series (6 trials in total). Each trial was randomly chosen between 16 and 50 s for the short interval durations, and between 2 and 6 min for the longer interval durations. The inter-trial intervals were randomly chosen between 5 and 15 s. After each temporal interval, the participants gave their verbal temporal judgment. Before the 30-min exercise, they were instructed that they would have to judge in seconds and then in minutes (and vice versa) the duration between the two beeps presented during the exercise (i.e. prospective judgment). They were also told that the short durations were between 1 and 80 seconds and the long ones between 1 and 12 minutes. They were also instructed not to interrupt the exercise and not to count time, in accordance with the procedure used to prevent counting strategies previously tested by Rattat and Droit-Volet [45]. A demonstration trial was given before the 30-min exercise. Finally, after the exercise (post-exercise), the participants filled in the scales again.

#### 2.1.6 Data analyses

Statistical analyses were performed using the IBM SPSS software (version 3.2). An analysis of variance (ANOVA) was run on the mean time estimates with the interval duration (seconds vs. minutes) as within-subjects factor and the meditation group (meditation vs. control) as between-subjects factor (Table D in S1 File). For the time estimates, we calculated the temporal standardized error, i.e. the difference between the temporal estimate and the interval duration divided by the interval duration. A standardized error greater than zero indicates a temporal overestimation and one smaller than zero a temporal underestimation. A series of ANOVAs was also carried out on each score on the various self-reported scales with the 3 testing times (pre-training, pre-exercise, post-exercise) as within-subjects factor and the meditation group as between-subjects factor. When a significant effect was observed, Bonferroni tests were used to examine the differences between two within-conditions.

After the analyses of variance, a correlation matrix was drawn up between the time estimates (standardized error) for the two temporal scales (seconds and minutes) and the scores on the different scales (anxiety, happiness, PoT, present-moment awareness) (with all subjects collapsed into one group). In the case of significant correlations, we examined the mediating effect of scale scores on the significant relationship between the meditation exercise and time estimates, as well as on the judgment of the passage of time. The bootstrapping mediating method was applied by using the SPSS process macro written by Hayes [46]. 5000 bootstrapping was used to identify indirect effects in the mediating models, with 95% confidence interval (95% CI). Coefficients were considered significant (*p* < .05) when 95% CI did not cross zero. Nevertheless, we calculated the z-value and p-value of the indirect effect using the medmod module of the jamovi software [47].

### 2.2 Results

#### 2.2.1 Temporal estimates

**Fig 2:**
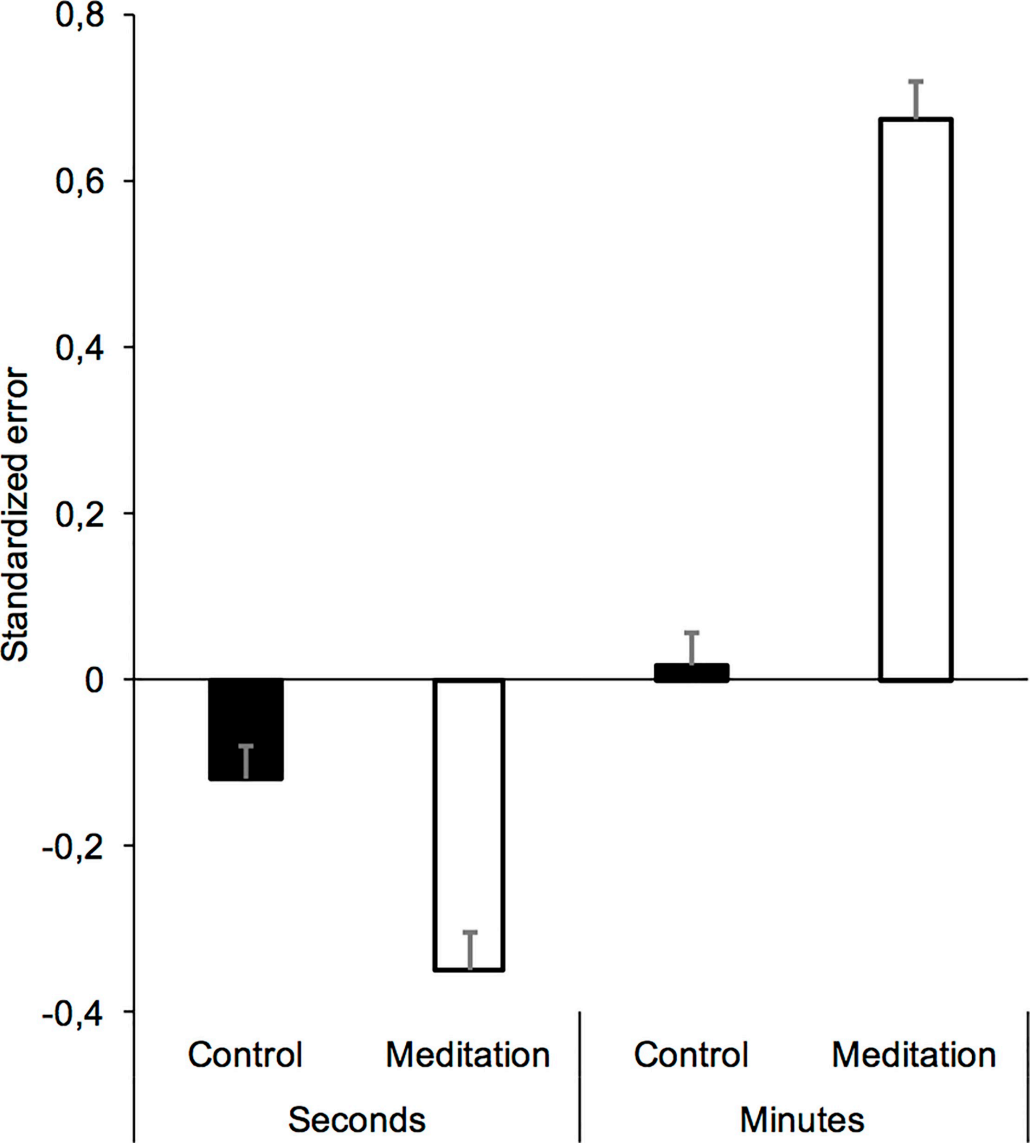
Time estimates. Mean temporal standardized error for the two ranges of interval durations (seconds and minutes) in the meditation and the control group (*bar errors = standard errors*).

Fig 2 shows the mean of the standardized errors for the two ranges of interval durations (seconds vs. minutes) in the meditation and the control group (Table A in S1 File). The ANOVA performed on this temporal standardized error showed a significant interaction between interval duration and meditation group, *F*(1, 58) = 94.93, *p* < 0.001, *n*^*2*^_*p*_ = 0.62, which subsumed significant main effects of interval duration, *F*(1, 58) = 161.92, *p* < 0.001, *n*^*2*^_*p*_ = 0.74, and meditation, *F*(1, 58) = 30.63, *p* < 0.001, *n*^2^_p_ = 0.34. We therefore conducted statistical analyses for each temporal range taken separately.

There was a significant effect of meditation for the interval durations in the seconds and the minutes range (*F*(1, 58) = 17.57, *p* < 0.001, *n*^*2*^_*p*_ = 0.23; *F*(1, 58) = 105.07, *p* < 0.001, *n*^2^_*p*_ = 0.64). However, the interval durations were judged shorter by the meditation group (M = -0.35, SD = 0.27) than by the control group (M = -0.12, SD = 0.13) in the second-duration range, whereas they were judged longer by the meditation group (M = 0.67, SD = 0.33) than by the control group (M = .016, SD = 0.12) in the minute-duration range. The direction of the time distortions was thus inverted at the different temporal scales. Indeed, the *t*-test comparing the standardized error to zero confirmed that the interval durations were significantly underestimated at the temporal scale of seconds and significantly overestimated at that of minutes in the meditation group (*t*(29) = -7.014, *t*(29) = 11.17, respectively, both *p* < .0001). In the control group, the temporal intervals were also underestimated for short durations, although to a lesser extent, *t*(29) = -5.15, *p* < .0001, and were close to zero for the longer durations, *t*(29) = 0.72, *p* = .48.

#### 2.2.2 Anxiety

Table 1 shows the mean and standard deviation of scores obtained in the meditation and the control group on the 4 self-reported scales (anxiety, happiness, passage of time, present-moment awareness) for the 3 testing times, i.e. before the training, and just before and after the exercise.

**Table 1:** Scores on the self-reported scales.

	Anxiety		Happiness		PoT		Present	
	M	SD	M	SD	M	SD	M	SD
***Meditation***								
**Pre-training**	20.49	16.48	108.52	28.74	7.67	2.94	5.99	3.99
**Pre-exercise**	13.00	10.09	125.04	23.72	8.17	3.013	7.82	3.36
**Post-exercise**	5.72	6.39	152.26	16.79	12.16	1.49	12.02	2.31
***Control***								
**Pre-training**	25.54	12.17	109.76	31.41	5.19	2.83	8.65	3.13
**Pre-exercise**	27.03	9.45	110.13	28.78	5.007	2.69	8.61	2.84
**Post-exercise**	25.12	9.17	110.50	30.98	2.41	1.217	9.98	2.89

Scores in cm (14-cm line) for the PoT and the Present-moment awareness question, and sum of scores in cm of different items for the anxiety and happiness scale

The ANOVA on the anxiety scores showed a significant interaction between the meditation group and the testing time, *F*(2, 116) = 16.46, *p* < 0.001, *n*^*2*^_*p*_ = 0.22, with a significant main effect of meditation, *F*(1, 58) = 27.23, *p* < 0.001, *n*^2^_p_ = 0.32, and testing time, *F*(2, 116) = 18.30, *p* < 0.0001, *n*^*2*^_*p*_ = 0.24. In the control group, there was no significant difference in anxiety scores between the three testing times, *F*(2, 58) = 1.06, *p* = 0.35. In contrast, in the meditation group, the anxiety scores decreased across the testing times, *F*(2, 58) = 24.25, *p* < 0.001, *n*^*2*^_*p*_ = 0.46, decreasing from the pre-training to the pre-exercise test (20.49 vs. 13.00, Bonferroni test, *p* = 0.002), and from the pre-exercise to the post-exercise test (13 vs. 5.72, *p* < 0.0001). Anxiety was therefore significantly lower at the post-exercise test in the meditation than in the control group, *t*(58) = 9.51, *p* < .0001.

#### 2.2.3 Happiness

For the happiness scores, there was also a meditation x testing time interaction, *F*(2, 116) = 39.79, *p* < 0.001, *n*^2^_p_ = 0.40, with a significant meditation, *F*(1, 58) = 8.23, *p* < 0.006, *n*^2^_p_ = 0.12, and testing time effect, *F*(2, 116) = 42.51, *p* = 0.0001, *n*^2^_p_ = 0.42. The feeling of happiness did not change in the control group, *F*(2, 58) = 0.04, *p* = 0.97, whereas it increased across the tests in the meditation group, *F*(2, 58) = 60.42, *p* < 0.0001, *n*^2^_p_ = 0.68. Indeed, the participants reported being happier after meditation training (pre-training *vs*. pre-exercise) and a long meditation exercise (pre-exercise *vs*. post-exercise) (both Bonferroni tests, *p* < 0.001). Consequently, the happiness level was higher in the meditation than in the control group at the post-exercise test, *t*(58) = 6.59, *p* < .0001.

#### 2.2.4 Passage-of-time judgment

The ANOVA on the score (cm) for the PoT judgment found a meditation x testing time interaction, *F*(2, 116) = 59.09, *p* < 0.0001, *n*^2^_p_ = 0.51, with a significant effect of meditation group, *F*(1, 58) = 116.14, *p* < 0.0001, *n*^2^_p_ = 0.67, and of testing time, *F*(2, 116) = 3.04, *p* = 0.05, *n*^2^_p_ = 0.052, although this latter effect only just reached significance. The PoT judgment varied across the testing phases for both the control and the meditation group (*F*(2, 58) = 23.97, *p* < 0.0001, *n*^2^_p_ = 0.45; *F*(2, 58) = 35.21, *p* < 0.0001, *n*^2^_p_ = 0.55, respectively). The passage of time was nevertheless judged to be slower at post-exercise testing than at pre-exercise testing in the control group (2.41 vs. 5.01, Bonferroni tests, *p* < .0001), whereas it was judged to be faster at post- than at the pre-exercise testing in the meditation group (12.16 vs. 8.17, *p* < .001). The passage of time was thus experienced as being faster with a meditation exercise than a control exercise (12.16 vs. 2.41, *t*(58) = 22.67, *p* < .0001). No difference in the PoT judgment was observed between the pre-training and the pre-exercise test times for either the meditation or the control group (Bonferroni tests, all *p* < .05).

#### 2.2.5 Present-moment awareness

There was also a meditation x testing time interaction for present-moment awareness (scores in cm), *F*(2, 116) = 18.48, *p* < 0.0001, *n*^2^_p_ = 0.48, with a significant testing time effect, *F*(1, 58) = 116.14, *p* < 0.0001, *n*^2^_p_ = 0.67, but no significant meditation group effect, *F*(1, 58) = 0.50, *p* = 0.48. There was a significant effect of testing time in both the control and the meditation group (*F*(2, 58) = 5.77, *p* = 0.01, *n*^2^_p_ = 0.17; *F*(2, 58) = 48.09, *p* < 0.0001, *n*^2^_p_ = 0.62, respectively). In both groups, the participants were more aware of the present-moment after than before a 30-min exercise (Bonferroni, *p* < .01). However, present-moment awareness was higher at post-exercise test time in the meditation group than the control group (12.07 vs. 9.98, *t*(58) = 3.01, *p* = .004). Moreover, present-moment awareness increased from pre-training to pre-exercise in the meditation group (*p* = .01), while it did not change with training in the control group (*p* > .05).

#### 2.2.6 Correlations between temporal estimates and self-reported experiences

Table 2 shows the correlation between the temporal standard errors for the interval durations in the seconds and the minutes range, and the scores on each self-reported scale recorded after the 30-min exercise. An examination of Table 2 reveals a significant correlation between time estimates in the two temporal scales (r = -.47, *p* < .001). The underestimation of temporal intervals in the seconds range was therefore associated with an overestimation of temporal intervals in the minutes range. However, it appears that the experience of PoT, the anxiety and the happiness level were also correlated with the time estimates in the two duration conditions (all *p* < .05), whereas present-moment awareness was only significantly correlated with time estimates of long durations of several minutes. The more the participants were focused on the present moment, the longer their time estimates in the minutes time scale.

**Table 2:** Correlation matrix between the standardized temporal error in the seconds and the minutes range and the scores on the different scales.

	1	2	3	4	5	6
1. Temporal errors (sec)	1	-.47**	.36**	-.30*	-.45**	-.07
2. Temporal errors (min)	-.47**	1	-.63**	.55**	.75**	.30*
3. Anxiety	.36**	-63**	1	-.68**	-.74**	-.47**
4. Happiness	-.30*	.55**	-.68**	1	.62**	.37*
5. Passage of time	-.45**	.75**	-.74**	.62**	1	.35**
6. Present	-.07	.30*	-.47**	.31*	.35**	1

* p < .05;

** p < .01

Moreover, the scores on the different self-reported scales were significantly intercorrelated (Table 2). Therefore, we decided to examine the mediating effect of scores on each subjective scale in the significant relationship between the meditation exercise (meditation *vs*. control) and the time estimates (standardized temporal errors). Table 3 reports the indirect effect obtained with the Bootstrap mediating analyses. The meditation exercise always explained the subjective experience reported in the different scales (all *p* < .0001). However, there was no significant indirect effect of anxiety, happiness, PoT judgment, or present-moment awareness on the relationship between the meditation exercise and the time estimates in the two temporal scales (all *p* > .05).

**Table 3:** Mediating indirect effects on the significant relationship between the mediation exercise and both the time estimates (standardized errors) in the seconds and the minutes range and the passage of time judgment. The bootstrapping mediating method developed by Hayes [46] was used with 95% confident intervals. Coefficients were considered significant when 95% CI did not cross zero (n = 60, 5000 bootstrapping).

	Ind. effect	BootSE	BootLLCI	BootULCI	*Z*	*p*
**Time estimates (sec)**						
Anxiety	0.0128	0.0779	-0.1356	0.176	0.16	.87
Happiness	0.0068	0.0556	-0.0952	0.1198	0.12	.90
PoT	0.1232	0.2832	-0.4159	0.6861	0.42	.67
Present	0.0219	0.0192	-0.0093	0.0649	1.14	.26
**Time estimates (min)**						
Anxiety	-0.0013	0.0534	-0.125	0.0936	0.02	.98
Happiness	0.0245	0.348	-0.0444	0.0945	0.70	.49
PoT	-0.2716	0.2369	-0.7474	0.1935	1.15	.25
Present	0.0032	0.0221	-0.0433	0.0465	0.15	.88
**Passage of Time**						
Time esti. (sec)	-0.1192	0.3046	-0.6573	0.5658	0.40	.69
Time esti. (min)	-0.554	0.4689	-1.4516	0.401	1.23	.22
Anxiety	-0.1623	0.4709	-1.1612	0.7348	0.35	.73
Happiness	-0.0201	0.3504	-0.752	0.6211	0.07	.95
Present	-0.0344	0.1259	-0.3189	0.1921	0.27	.79

LLCI = lower limit confidence interval; ULCI = Upper limit confident interval

#### 2.2.7 Correlations between passage-of-time judgment and self-reported experiences

The passage of time judgment was also significantly related to all the other dimensions (Table 2). The passage of time was judged to slow down when the level of anxiety or sadness increased. It also slowed down when the participants were less focused on the present-moment. The mediating effects of self-reported scores on the association between the meditation exercise and the PoT judgment were then measured in the same way as for the duration judgments (Table 3). The results indicated that anxiety, happiness and present-moment awareness did not significantly mediate the significant relationship between the meditation exercise and the PoT judgment. The estimations of interval durations during the exercise also did not predict the differences in the subjective experience of the passage of time. Only the meditation exercise explained the variance in the PoT judgment.

### 2.3 Discussion

The results of our experiment showed that a long exercise of mindfulness meditation and a daily training program significantly reduced anxiety and increased the feeling of happiness. These results provide additional data for the growing database demonstrating the beneficial effect of mindfulness meditation on affectivity [48], even in the case of only brief mindfulness training [49]. They also demonstrate the efficacy of the procedure used in our experiment, since the meditation exercise induced the expected effects on happiness and anxiety. They also demonstrate that time judgments are highly sensitive to meditation effects. However, our results revealed that the time judgment in a meditation exercise differed as a function of the time scale, depending on whether this was in the range of seconds or minutes. Indeed, the interval durations from 15 to 50 sec were underestimated in the meditation condition compared to the control condition, whereas those from 2 to 6 min were overestimated. In addition, the temporal shortening found for the seconds range was significantly correlated with the temporal lengthening found for the minutes range: the shorter the estimated time for the durations of several seconds, the longer the estimated time for the durations of several minutes. This reversal in the direction of the time judgment for the different temporal scales suggests that different processes might underlie the judgment of short and long durations in the same meditation condition.

The meditation-related temporal underestimation in the seconds time scale obtained in our study is consistent with the results of studies on meditation showing that experienced meditators or students trained to practice meditation underestimate short durations during a meditation exercise. Indeed, similar temporal underestimations have been found with interval durations of 15, 30 and 60 sec in two recent experiments carried out by Droit-Volet et al. [16] with students who were or were not trained in meditation, and with different mindfulness meditation techniques (body-scan, breathing). Similar temporal underestimations have also been found in students and experienced meditators when judging short stimulus durations (from 0.3 to 2.5 s) administered during the mindfulness exercise [22]. As discussed in the Introduction, the internal clock models explain this shortening of time estimates in terms of attention mechanisms [30]. Indeed, numerous studies using the dual-task paradigm have shown that estimated durations shorten when attentional resources are distracted away from time processing. The shortening of time in response to a meditation exercise thus results from the amount of attentional resources devoted to the meditation exercise to the detriment of attentional resources allocated to time processing.

The idea that an attention-based mechanism may be responsible for the shortening of time produced by a meditation exercise cannot be logically used to explain the lengthening of time observed with long interval durations in our study. Unlike our results on the effect of a meditation exercise on the judgment of short durations, those on the judgment of long durations of several minutes are totally new. In addition, only a few studies have experimentally examined the judgment of long durations of several minutes. Nevertheless, as suggested in the Introduction, the judgment of long durations is necessarily based on long-term memory processes [39]. In the studies on the retrospective judgment of durations, estimated duration depends on the content of the interval to be judged, namely the characteristics of events (number, complexity) or the activities performed during this interval (effortless, attentionally demanding) [33,34]. According to the storage-size model of retrospective time judgment, time estimates increase with the amount of storage available in memory [32]. We can thus assume that, in our study, the judgment of interval durations of several minutes was also based on memory processes close to those used in the retrospective time judgment. The aim of our second experiment was therefore to replicate our original findings on the effect of a meditation exercise on the judgment of long durations of several minutes and to examine the link between time estimates and the memory load resulting from the attentional demand of the meditation exercise and/or its perceived difficulty.

The direction of the time distortions (shortening *vs*. lengthening) produced by a meditation exercise was found to change depending on the temporal scale considered. However, whatever the direction of the time distortion, the participants always reported that time passed faster with a meditation exercise. They said that time went faster, while producing shorter time estimates in one case and longer time estimates in the other. As further discussed later, this dissociation between the judgment of the passage of time and the interval duration judgments suggests that these two forms of temporal judgment are completely, or at least partially, different. In particular, our results showed significant correlations between the PoT judgment and the duration judgment, but the PoT judgment did not mediate the effect of meditation on duration judgments. Using an ecological momentary assessment method in which the PoT judgment and the duration judgment were measured at the same time, Droit-Volet and her colleagues effectively found that the PoT judgment did not predict the judgment of short durations [33,38]. At the same time, however, they found that it predicted the judgment of long durations such as those used in our study. In our experiment, the participants judged the passage of time for the entire duration of the 30-min exercise, while they judged the duration of the interval that had just elapsed in the other studies. The different time periods evaluated in the case of duration judgments and PoT judgments could thus explain why our results for the long durations were different from those obtained by Droit-Volet et al. [33,38] with the ecological momentary assessment method. Therefore, in Experiment 2, we examined these two forms of temporal judgment for the recently elapsed interval duration (i.e., after each trial) in the case of long durations of several minutes, to further examine our original results found with long durations.

In Experiment 2, new participants, also trained to practice mindfulness meditation, performed a 30-min body-scan mindfulness exercise or a control exercise. They had to judge interval durations of several minutes presented during these exercises as in Experiment 1. However, after each interval duration, they also had to judge the PoT as well as the attentional demand, the difficulty of the exercise, the degree of focus on the present-moment, and the arousal level. Our hypothesis was that the interval durations of several minutes would be overestimated in the meditation compared to the control condition, and this temporal overestimation would be significantly related to the attentional demand, the task difficulty or the focus on the present moment induced by the meditation exercise, as predicted by the retrospective memory models of time.

## 3 Experiment 2

### 3.1 Method

#### 3.1.2 Participants

Thirty new undergraduate students, who received course credits, took part in this experiment (9 men; 21 women; Mean age = 26.60, SD = 5.68). They signed a written consent form containing the description of the procedure, which had been approved by the Sud-Est VI statutory Ethics committee (CPP), according to the French law.

#### 3.1.3 Material and procedure

The materiel was the same as that used in Experiment 1 (Fig 1). The procedure was also very similar, with a daily meditation training phase on each of 7 days. However, in Experiment 2, each participant performed both the meditation exercise and the control exercise (listening to poems). Two 33-minute exercises were performed the same day after a 15-minute break, and when the participants judged that they were ready for the second exercise. The exercise order was counterbalanced across subjects. During each exercise, there were 4 inter-beep interval durations to be judged (4 trials). Each interval duration was successively presented in a window of 7.5 minutes, with a 1-min inter-window interval (Fig 1). The interval durations were randomly chosen between 2 and 6 minutes. The participants were told that the interval durations would be between 1 and 12 minutes and that they should not interrupt the exercise. The originality of the procedure used in Experiment 2 was that the participants not only gave their verbal judgment of the interval duration that had elapsed, but also their judgment for 5 other dimensions: (1) passage of time, (2) attention, (3) difficulty, (4) arousal, (5) present-moment. They thus answered 5 questions: “during this inter-beep duration, how much, as a percentage, do you think that (1) time has passed faster compared to a clock, (2) this exercise has consumed your attentional resources, (3) this exercise was difficult, (4) you were more calm/relaxed or excited/awake, (5) your awareness was focused on the present-moment. The participants answered orally by given a percentage between 0 and 100%. Their oral responses were recorded by computer. In addition, the different questions and response scales were presented before the exercise in such a way that only the title of the question was given during the exercise. The presentation order of these 5 questions was random.

In addition, the participants filled in the self-reported scale of anxiety (STAI: pre-control exercise, α = .968; post-control exercise, α = .972; pre-meditation exercise, α = .958; post-meditation exercise, α = .978) and happiness (SA-DHS: pre-control exercise, α = .986; post-control exercise, α = .992; pre-meditation exercise, α = .98; post-meditation exercise, α = .958) before (pre-exercise) and after (post exercise) each exercise. As indicated by Cronbach’s alpha, the reliability of these scales was satisfactory.

#### 3.1.4 Data analyses

An initial repeated measures analysis of variance was performed on the scores of anxiety and those of happiness, which were assessed before and after each exercise in order to verify the effectiveness of the meditation exercise on these important criteria. Three within-subjects factors were tested: meditation (meditation *vs*. control exercise), testing times (pre- *vs*. post-exercise) and session order (meditation-control *vs*. control-meditation).

To facilitate the comparison between the results of Experiments 1 and 2, we decided to initially perform analyses of variance on the mean time estimates (temporal standardized error) and the mean score on each of the other dimensions (PoT, attentional demand, task difficulty, arousal level and focus on the present moment), with the meditation condition as within-subjects factor. However, as there were multiple non-independent responses for the same subject for each trial (temporal interval randomly chosen), we also ran a linear mixed model (LMM) to verify the relationships between our data (Table E in S1 File). The LMM is a form of linear regression which allows us to test whether one variable (X) is a significant predictor of another outcome variable (Y). We thus conducted two series of analyses: the first on the time estimates and the second on the PoT judgment as dependent variable, with the meditation exercise and each dimension entered separately as fixed factor into the model, and with random effects for the subjects and the trials. Then, we examined whether each significant dimension predicting time estimates and PoT judgment found by the LMM had a significant mediating effect on the significant relationship between the meditation exercise and temporal performance. As we have multilevel data, we used the ml_mediation program of the Stata 15 software, adapted from Krull and Mackinnon [50], with 500 bootstrapping.

The exercise order factor was excluded from the statistical analyses because initial analyses indicated that this factor was not significant for any of the judgments produced during the 30-min exercise (interval duration judgment, PoT, attention, difficulty, arousal, present-moment, (all *p* > .05).

### 3.2 Results

#### 3.2.1 Anxiety and happiness

The ANOVAs showed a significant meditation x session order interaction for both the anxiety and the happiness scores (*F*(1, 28) = 41.67, *p* < 0.001, *n*^2^_p_ = 0.598; *F*(1, 28) = 61.85, *p* < 0.001, *n*^2^_p_ = 0.69). When the participants performed the control exercise first, there was no difference in the anxiety and the happiness scores at pre-exercise testing between the two sessions (28.90 *vs*. 27.50, *t*(14) = 1.56, *p* = .14; 102.48 *vs*. 103.17, *t*(14) = -.26, *p* = .80, respectively). However, when the meditation exercise was performed first, the anxiety scores were lower and the happiness scores higher for pre-exercise testing in the second than in the first session (9.07 *vs*. 17.66, *t*(14) = -6.192, *p* > .001; 142.21 *vs*. 114.21, *t*(14) = 7.38, *p* > .001). This indicates the “long-term” influence of an initial meditation exercise.

However, there was a significant interaction between meditation and the testing time for both the anxiety and the happiness scores, with no other significant interaction (*F*(1, 28) = 94.71, *p* < 0.001, *n*^2^_p_ = 0.77; *F*(1, 28) = 215.94, *p* < 0.001, *n*^2^_p_ = 0.89, respectively). There was indeed no change in anxiety and happiness before and after the control exercise (18.99 *vs*. 18.21, *t*(29) = 1.65, *p* = .11; 122.35 *vs*. 124.52, *t*(29) = -1.49, *p* = .15, respectively), while anxiety was significantly lower and happiness higher after than before the meditation exercise (22.58 *vs*. 8.77, *t*(29) = 8.89, *p* < .001; 108.69 *vs*. 146.11, *t*(29) = -19.37, *p* < .0001), and this whatever the session order. As in Experiment 1, the meditation exercise therefore had the expected effects on the individual levels of anxiety and happiness, by decreasing and increasing them, respectively.

#### 3.2.2 Temporal estimates

**Fig 3:**
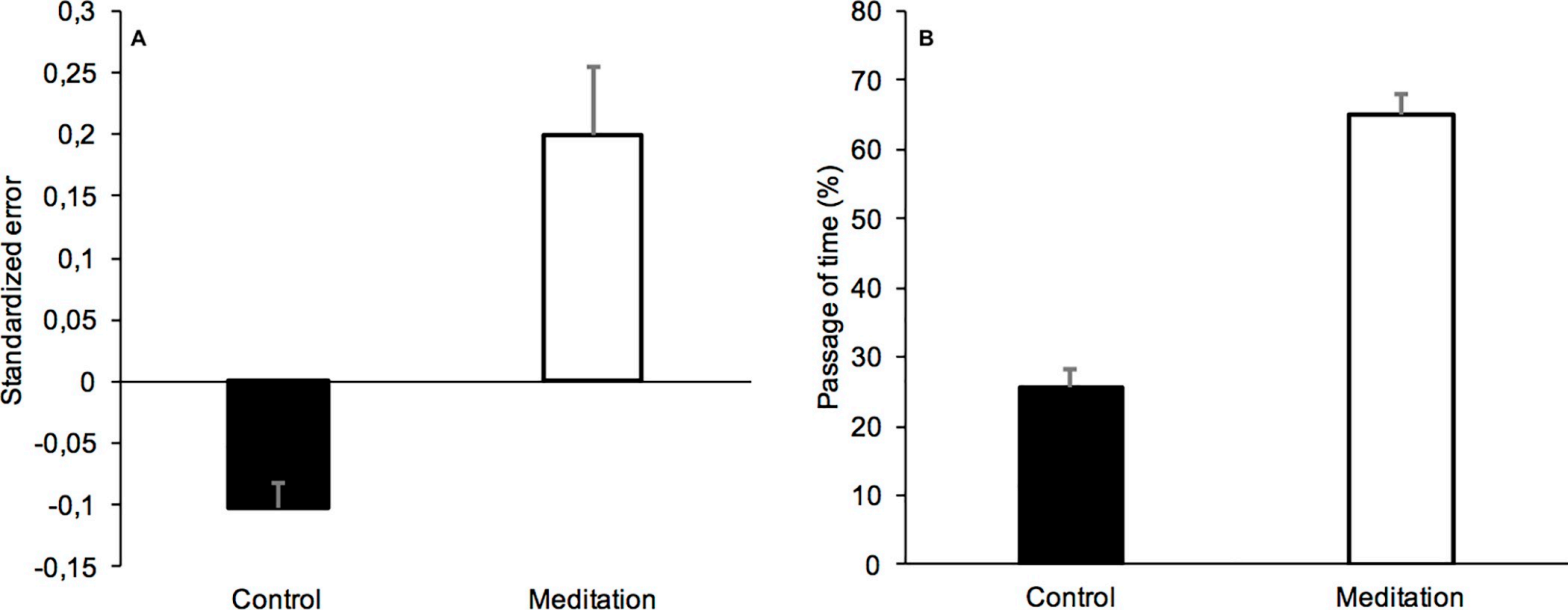
Time estimates and passage of time judgment. (A) Mean (*SE*) temporal standardized error and (B) mean (*SE*) passage of time judgment in the meditation and the control group for the minute duration range.

Fig 3A presents the mean temporal standardized errors for the meditation and the control exercise (Table B in S1 File). The ANOVA found a significant effect of meditation group, *F*(1, 29) = 26.75, *p* < 0.0001, *n*^2^_p_ = .48. These results therefore replicated those found in Experiment 1, showing that the long interval durations of several minutes were overestimated during the meditation exercise (M = 0.199, SD = 0.31) compared to the control exercise (M = -0.102, SD = 0.108).

**Fig 4:**
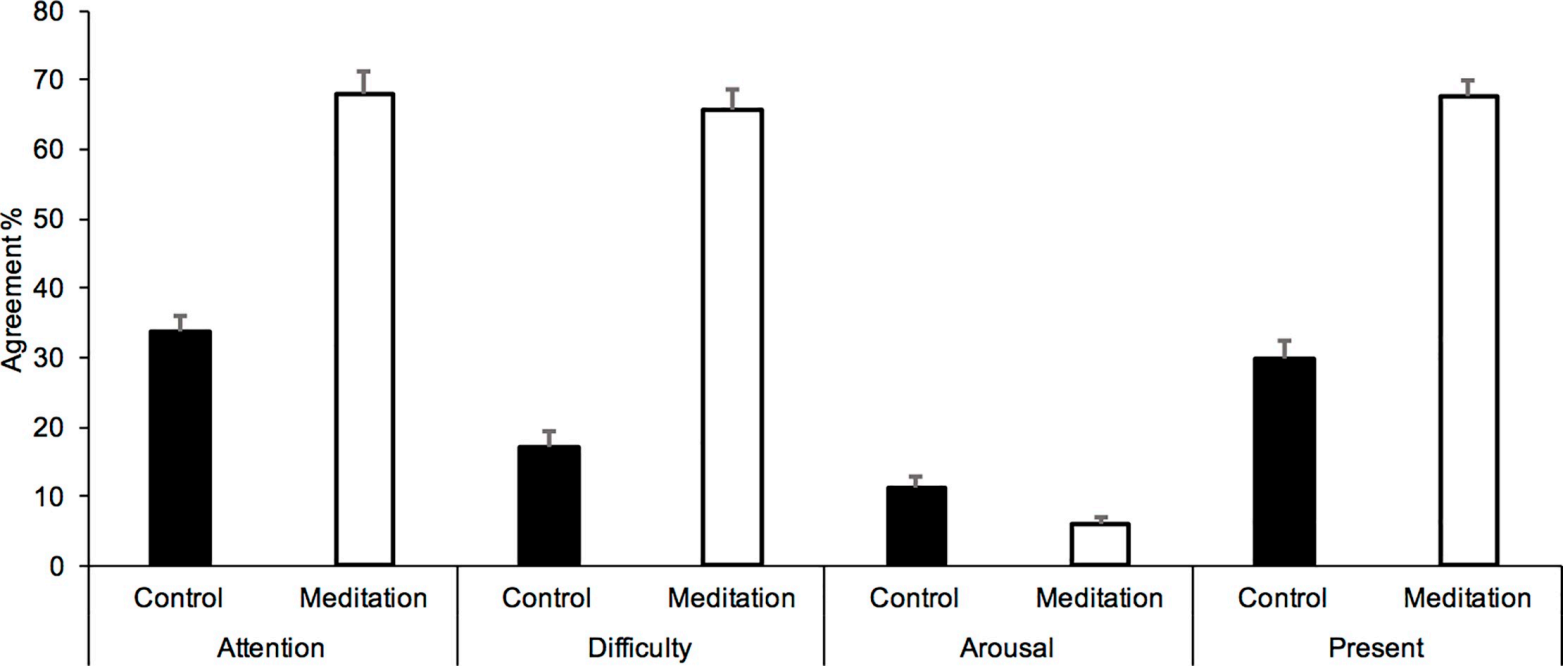
Judgment scores. Mean (*SE*) percentage of agreement for attentional demand, task difficulty, arousal and focus on the present moment in the meditation and the control condition.

However, the effect of meditation exercise was also significant on the scores (from 0 to 100%) for the other five judgments: (1) PoT (Fig 3B) (M_med_ = 65.21, SD = 15.68, M_cont_ = 25.58, SD = 14.03, *F*(1, 29) = 124.46, *p* < 0.0001, *n*^2^_p_ = .81), (2) attentional demand (M_med_ = 68, SD = 17.31, M_cont_ = 33.83, SD = 12.63, *F*(1, 29) = 137.05, *p* < 0.0001, *n*^2^_p_ = .83), (3) task difficulty (M_med_ = 65.54, SD = 17.03, M_cont_ = 17.08, SD = 11.85, *F*(1, 29) = 161.51, *p* < 0.0001, *n*^2^_p_ = .85), (4) focus on the present moment (M_med_ = 67.71, SD = 12.42, M_cont_ = 29.79, SD = 13.59, *F*(1, 29) = 219.03, *p* < 0.0001, *n*^2^_p_ = .88), and (5) arousal (M_med_ = 6.13, SD = 4.599, M_cont_ = 11.29, SD = 8.76, *F*(1, 29) = 27.76, *p* < 0.0001, *n*^2^_p_ = .49) (Fig 4) (Table C in S1 File).

The LMM confirmed the significant fixed effect of the meditation exercise on time estimates (b = 0.30, SE = 0.037, 95%CI[0.23; 0.37], *t* = 8.14, *p* < .0001). There was also a significant meditation effect on the scores of other self-reported dimensions (PoT, attentional demand, task difficulty, arousal level and focus on the present moment) (all *p* < .01, Table 4). In other words, the meditation exercise lengthened the time estimates but also increased the attentional demand, the task difficulty, and the focus on the present moment. They also decreased the arousal level.

**Table 4:** Fixed effect of judgments on the temporal standardized error or the passage of time judgment using the Linear mixed model.

	Estimate	SE	LLCI	ULCI	*t*	*p*-value
**Fixed effect on temporal error**						
Meditation	0.3014	0.0370	0.2281	0.3747	8.140	< .0001
Passage of time	0.0037	0.00079	0.0021	0.0052	4.622	< .0001
Attention	0.0050	0.00084	0.0033	0.0066	5.900	< .0001
Difficulty	0.0037	0.00070	0.0023	0.0051	5.328	< .0001
Present	0.0068	0.0007	0.0054	0.0083	9.189	< .0001
Arousal	-0.0071	0.0026	-0.0122	-0.0019	-2.712	< .01
**Fixed effect on the passage of time**						
Meditation	39.625	1.9203	35.822	43.437	20.64	< .0001
Temporal error	22.434	4.8533	12.873	31.995	4.622	< .0001
Attention	0.5138	0.0622	0.3913	0.6364	8.26	< .0001
Difficulty	0.5633	0.0441	0.4761	0.6505	12.77	< .0001
Present	0.6899	0.0525	0.5864	0.7934	13.13	< .0001
Arousal	-0.7468	0.2016	-1.1439	-0.3498	-3.705	< .0001

Therefore, we examined the mediating effect of each self-reported dimension in the significant relationship between the meditation exercise and the time estimates (Table 5). These analyses indicated that the PoT judgment, the attentional demand, the task difficulty and the arousal level experienced by the participants did not mediate the relationship between the meditation exercise and the time estimates (both *p* > .05). However, there was a significant indirect effect of the focus on the present moment (b = 0.247, SE = 0.07, 95%CI [0.10; 0.39], z = 3.39, *p* < .05). Indeed, the meditation factor lost its significance on time estimates when present-moment awareness was included in the model (b = 0.05, SE = 0.06, 95%CI [-0.069; 0.177], z = 0.86, *p* > .05. Therefore, present-moment awareness was a significant mediator of the total effect of the meditation exercise on the time estimates for long intervals of several minutes. This suggests that the effect of the meditation exercise in terms of overestimation of interval durations of several minutes was mainly related to the self-awareness to be focused on the present moment. The more the participants reported being focused on the present moment, the longer they judged the interval durations to be. However, present-moment awareness and the attention devoted to the exercise were closely linked (b = 0.598, SE = 0.05, 95%CI [0.49; 0.70], z = 11.39, *p* < .05). The more attentional resources the exercise consumed, the more focused on the present moment the participants reported themselves to be.

**Table 5:** Mediating indirect effects on the association between the mediation exercise and the time estimates (standardized errors) or the passage of time judgment.

	Ind. effect	BootSE	BootLLCI	BootULCI	*Z*	*p*
**Time estimates (min)**						
PoT	-0.137	0.093	-0.3197	0.0447	-1.48	.14
Difficulty	-0.097	0.121	-0.3354	0.1407	-0.80	.42
Attention	-0.047	0.085	-0.112	0.2136	0.56	.58
Present	0.247	0.073	0.1043	0.3905	3.39	.001
Arousal	0.0188	0.0192	-0.0188	0.0564	0.98	.33
**Passage of Time**						
Time estimates	-2.712	1.426	-5.507	0.084	-1.90	.057
Difficulty	-1.853	8.851	-19.201	15.485	-0.21	.83
Attention	-7.674	5.875	-19.190	3.841	-1.31	.19
Present	0.440	5.448	-10.128	11.118	0.08	.94
Arousal	-0.899	0.988	-2.835	0.1037	-0.91	.36

LLCI = lower limit confidence interval; ULCI = Upper limit confident interval

#### 3.2.3 The passage of time judgment

As reported above, the statistical analyses performed using both an ANOVA and a LMM showed a significant effect of the meditation exercise on the PoT judgment (both *p* < 0.0001), indicating that the participants reported an acceleration of the passage of time in the meditation compared to the control condition (65.21 *vs*. 25.58), while they overestimated the duration filled by the practice of meditation (Table 4, Fig 3B).

The LMM indicated that the fixed effects of the other dimensions on the PoT judgment were also significant (all *p* < .0001, Table 4), with the result that the feeling of the acceleration of the passage of time grew with increased attentional demand, task difficulty and attentional focus on the present. A subjective acceleration of passage of time was also observed when the level of arousal decreased (*p* < .0001). As in Experiment 1, the judgment of interval durations was also significantly linked to the PoT judgment for interval duration of several minutes (*p* < .0001): the longer the estimate of the interval duration, the faster the passage of time was judged to be.

The mediating analyses (Table 5) suggested that only the verbal estimation of interval duration tended to play a mediating role on the total effect of the meditation exercise on the PoT judgment (b = -2.71, SE = 1.426, 95%CI [-5.507; 0.84], z = -1.90, *p* = 0.057). The indirect effect of attentional demand did not reach significance (b = -7.67, SE = 5.87, 95%CI [-19.19; 3.84], z = -1.31, *p* < .05). However, when the attention factor was included in the model along with the meditation factor (equation 3, ml_mediation), it remained significant (b = -0.22, SE = 0.072, 95%CI [-0.365; -0.083], z = -3.12, *p* = 0.002), as also did the meditation effect factor (b = 47.30, SE = 2.89, 95%CI [41.63; 52.97], z = 16.35, *p* = 0.0001). Therefore, the participants’ feeling that the meditation exercise consumed their attentional resources partially contributed to the effect of the mediation exercise on the feeling that time passed faster.

## 4 General discussion

Experiments 1 and 2 showed a reduction in self-reported measures of negative affects (anxiety, sadness). This affective improvement appeared immediately after a meditation exercise, but also in the longer term as indicated by the significant effect of our daily-mindfulness training. These findings are consistent with the outcomes reported in the mindfulness training literature showing that the reduction of negative affects (fear, anxiety, sadness, depressive symptoms) is one of the main effects of mindfulness training [48]. The long-term benefits might be higher with 8–10 weeks of training than after a short period of training (7 days) such as that used in our study. A recent meta-analysis nevertheless testifies to the significant effect of brief mindfulness interventions on affects [49]. In addition, the length of a brief daily home training program did not moderate, or moderated only slightly, the effect on affects [49]. As suggested by Nair et al.’s study [51], it is possible to move rapidly into a reliable state of meditation after only a short period of meditation practice. Our studies also suggested that the immediate positive effects of a mindfulness exercise were related to the regulation of emotion (decrease in anxiety and arousal level, increase in happiness), as well as to the activation of attention control mechanisms [52,53,54]. The participants indeed reported that the mindfulness exercise placed them in the present-moment, was attentionally demanding and imposed an information processing load due to its difficulty. This is consistent with studies showing that mindfulness exercises facilitate attentional focus and reduce distracting thoughts such as mind-wandering and rumination [55,56,57]. However, the true originality of our studies lies in examining the effects of a mindfulness exercise and its underlying processes (attention control, emotion) on different types of time judgments, both the judgment of interval durations and that of the passage of time.

Our results showed that the interval durations of several seconds (from 16 to 50 sec) were underestimated with a mindfulness exercise compared to a control exercise. As already reported, these results replicated those found in a similar task of interval duration judgment with two different types of mindfulness techniques (body scan, breathing) and different activities used as a control task [16]. They also replicated those found in a task of stimulus duration judgment in both experienced meditators and participants with no prior meditation experience [22]. The shortening of durations in the seconds range in a mindfulness task is therefore a robust result. As stated in the Introduction, this shortening of time has been explained in the framework of predictions of attention-clock models that have been widely empirically validated [27,28]. According to these models, the judgment of durations directly depends on the amount of attentional resources allocated to time processing. It is thus likely that the shortening of short durations observed in our study with the meditation exercise results from the fact that the attentional focus is directed on the meditation activity at the expense of time processing. The smaller the quantity of attentional resources allocated to time processing, the shorter the perceived duration is. Obviously, a mindfulness exercise cannot be reduced to an attention task, as its effects on affects suggest. It is nevertheless an attention control task that consumes cognitive resources, as evidence by the statements of our participants in Experiment 2. In addition, numerous studies have demonstrated that regular practice of this type of mindfulness exercise increases attention skills because people train their attention control activity [58].

However, the temporal shortening observed for the estimation of short interval durations was not observed for that of longer interval durations of several minutes. Instead, the direction of the time distortion was reversed, i.e. a temporal lengthening instead of a temporal shortening. Indeed, in our study, the interval durations of several minutes were clearly overestimated in the meditation exercise compared to the control exercise. In addition, this temporal overestimation was observed in Experiment 1 and replicated in Experiment 2, attesting to the consistency of this result. To better understand this lengthening of time at the time scale of minutes, we questioned the participants on their experience, during the temporal interval that had elapsed, in terms of attention, task difficulty, focus on the present and arousal level. The statistical analyses revealed that the temporal lengthening of long durations produced by the meditation exercise compared to the control exercise was significantly linked to self-reported measures of attention (attention, difficulty) and focus on the present-moment. The more attentionally demanding and requiring a focus on the present the meditation exercise was judged to be, the longer the time estimates were. However, the mediation analysis revealed that only the self-reported assessment of being focused on the present moment significantly mediated the relationship between the meditation exercise and the time estimates. However, present-moment awareness and the attentional demand of the exercise were closely related. As indicated by our results, the scores on the attentional-demand scales were significant predictors of scores on the present-moment awareness scale. This suggests that time estimates at the temporal scale of minutes depend on the attentional focus on the present moment induced by the exercise. In the memory-based models, similar time extensions observed in retrospective time judgments are explained in terms of the size of memory storage [32,35,36]. The idea is that the retrospectively judged duration is a function of the amount of information stored during the temporal interval (amount of information, information complexity). Since then, it has been suggested that the segmentation of activity or the number of perceived changes also play a role [36]. As explained by Block [37], there is a long list of contextual factors that may influence the retrospective time judgment. Whatever these factors, the retrospectively judged duration depends on the non-temporal content of the interval to be timed, such as the workload involved in processing non-temporal information: the higher it is, the longer time is considered to be. Consequently, we may assume that the mindfulness meditation exercise had the same attentional impact on the participants in all conditions, but that this led to opposite results depending on the temporal scale, because the processing of long durations is largely dependent on a cognitive memory mechanism similar to those observed in the retrospective time judgment, and the processing of short duration is dependent on an internal clock system, whose operation requires attention. This provides additional support for the idea that the processing of short and that of long durations do not share the same mechanisms [39]. Another unsolved question is that of the temporal point at which we move from one mechanism to another, or rather the point when the high-level cognitive processes take over from the lower-level processes, because a clock system and a non-temporal processor can operate in parallel [59]. Our study does not allow us to answer this question, but it suggests that the awareness of passage of time is related to present-moment awareness and contributes to duration judgment during high-level cognitive processing.

In Experiment 2, when the PoT judgment was assessed for each interval to be timed, it appeared to be a significant predictor of estimated durations in the range of minutes, and vice versa. The PoT judgment and the duration judgment are thus significantly linked for the judgment of long durations of several minutes. This is consistent with the studies using ecological momentary assessment methods that have shown that the PoT judgment is a significant predictor of long durations, but not of short durations going from a few milliseconds to several seconds [38,39]. Consequently, time awareness plays a critical role in the different forms of time judgments for long intervals of several minutes. In addition, in Experiment 2, for each interval duration, the participants had to describe their experiences while they were practicing the exercise requested. They stated that, in comparison to the control exercise, they were calmer during the meditation exercise, more focused on the present-moment, and that their attention was focused on this task, which they judged more difficult. The statistical analyses revealed that time was judged to pass faster when the participants felt calmer and when their attention was focused on the exercise and the present moment, the two being obviously linked. Therefore, the more attention was focused on the required exercise, the longer the interval dedicated to this exercise was considered to be, and the faster external time was judged to pass. However, the mediation analyses did not show any significant indirect effect of these different dimensions (attention, difficulty, present) on the relationship between the meditation exercise and the PoT judgment. Nevertheless, the self-reported level of attention allocated to the exercise significantly contributed to the total effect of the meditation exercise on the PoT judgment when it was included in the statistical model. Therefore, the subjective experience of an acceleration of time arises partially from the fact that the cognitive resources are fully occupied by the realization of the mindfulness activity. However, the effect of mediation on the feeling that time passes faster cannot be reduced to attentional effects. Other dimensions must be examined, such as the sense of self and of body [13].

Most meditators say that their sense of time is altered when they meditate because they are outside time, i.e. in a state of timelessness. Our studies provide empirical data suggesting that this phenomenological description of their relationship to time arises to a large extent from their introspective analysis of their own internal state (attentional focus on the present activity) during the meditation experience. This conscious analysis of their internal state is thus translated into a feeling of duration—“self-duration”- compared to the representation of “world-duration” (external time) [13,60]. This allows them to state that time goes faster, because time is outside their mind, their attention being focused on the present-moment. However, further investigations are now required in experienced meditators because a meditation exercise may produce for them a state different from that observed in participants who have received only a short period of training, although the results of several studies do not support this idea [19,20,21]. Other investigations are also required to test durations longer than those used in our study (> 6 min), because the temporal task administered during the exercise may perhaps have interfered with meditation performance, although the interference effects were reduced with the long interval durations of between 2 and 6 minutes used in our experiments. It is also important to try to better understand the links in our study between the focus on the present-moment, attention and task difficulty in the experiences reported by the participants. The feeling of being focused on the present-moment may go beyond a problem of attentional focus. Other investigations are indeed needed to examine other dimensions, such as the sense of self which also play an important role in the mindfulness experience, especially in experienced meditators. It is in fact difficult to identify the influence of one major dimension, as the general state of consciousness is altered by mindfulness meditation.

## Supporting information

S1 File Tables of data used for Figures and statistical analyses. (XLSX)
